# Vertical transmission of human African trypanosomiasis: Clinical evolution and brain MRI of a mother and her son

**DOI:** 10.1371/journal.pntd.0005642

**Published:** 2017-07-27

**Authors:** Kathleen Gaillot, Marie-Agnès Lauvin, Jean-Philippe Cottier

**Affiliations:** Department of Diagnostic and Interventional Radiology and Neuroradiology, Bretonneau Hospital, Regional University Hospital Center of Tours, Tours, France; University of California San Diego School of Medicine, UNITED STATES

## Case report

A 22-year-old female was referred to our hospital from another in May 2013 to investigate a probable leukodystrophy. She was a political refugee from the Democratic Republic of Congo. Before arriving in France in 2010, she had spent 3 years in Angola (Bengo). She was the mother of a 13-month-old son (born in France in April 2012 after normal pregnancy, and having never traveled).

She initially entered the hospital for agitation and drowsiness. Physical examination showed slowing of ideomotor function, general hypotonia, clonic limb movements, and increased deep tendon reflexes.

**Fig 1:**
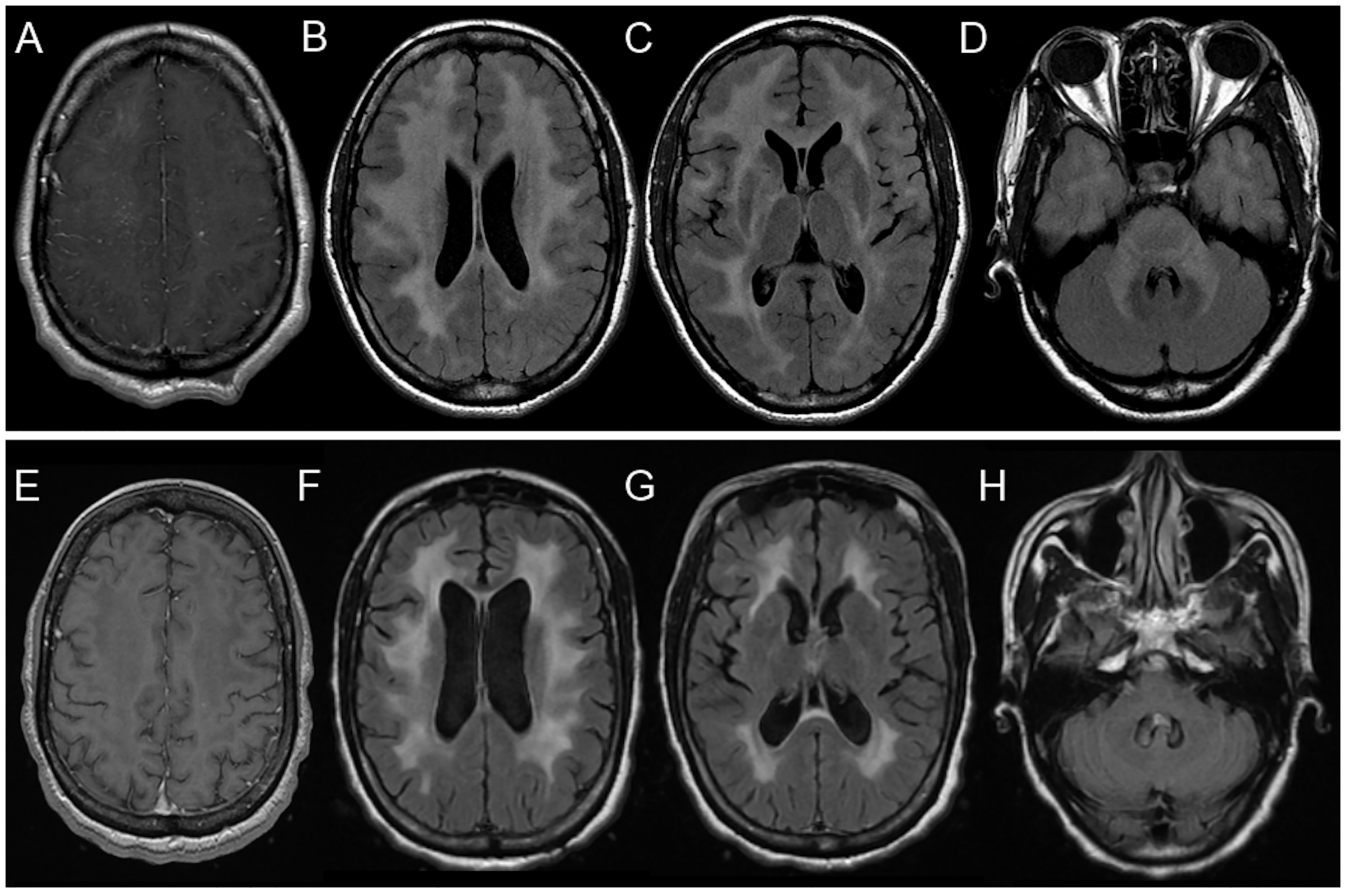
Brain MRIs of the mother before and after treatment of the meningoencephalitic stage of trypanosomiasis. (A) Initial brain MRI in January 2013 (axial T1-weighted image after injection of gadolinium chelates) showing perivascular and leptomeningeal enhancement, predominantly in the right corona radiata. (B, C, D) Initial brain MRI in January 2013 (axial fluid attenuated inversion recovery [FLAIR]-weighted images) showing extensive and confluent hyperintensities in both corona radiata and bilateral fronto-parieto-occipito-temporal white matter, internal and external capsules, lenticular nuclei, metencephalon, and middle cerebellar peduncles. Global brain atrophy with enlarged cerebrospinal fluid (CSF) spaces for a 22-year-old patient. (E) Control brain MRI in June 2014, 1 year after treatment (3D-T1 weighted image after injection of gadolinium chelates), showing resolution of the perivascular and leptomeningeal enhancement. (F, G, H) Control brain MRI in June 2014, 1 year after treatment (axial FLAIR-weighted images), showing resolution of the infratentorial and basal ganglia lesions, diminution of the supratentorial white matter lesions (especially capsular lesions), and increased secondary atrophy.

Brain MRI revealed bilateral T2 hyperintensities of the periventricular white matter (with perivascular and leptomeningeal enhancement), basal ganglia, and posterior fossa, with global brain atrophy for her age (Fig 1A–1D).

An electroencephalogram showed symmetrical frontotemporal theta and delta waves without paroxystic activity.

Blood analyses showed normochromic, normocytic, nonregenerative anemia related to minor iron deficiency (9.5 g/dL Hb, mean cell volume of 81 fL, 31.6 g/dL mean corpuscular hemoglobin, 28 G/L reticulocytes, normal hemoglobin electrophoresis), neutropenia (4.2 G/L leukocytes, 1.36 G/L neutrophil granulocytes, 0.14 G/L eosinophil granulocytes, 0.02 G/L basophil granulocytes, 2.16 G/L lymphocytes, and 0.53 G/L monocytes). Cerebrospinal fluid (CSF) analysis revealed lymphocytic pleiocytosis (280 white cells/μL, 90% lymphocytes), a normal glucose level (3.29 mmol/L), elevated lactate (2.65 mmol/L), protein (0.73 g/L), and IgG (0.272 g/L) levels, and an elevated IgG index (2.94). Investigations for infection were negative for tuberculosis (negative PCR and cultures on serum and CSF), herpes simplex virus (HSV) (PCR on CSF), HIV, viral hepatitis B and C, cytomegalovirus (CMV), human herpes virus (HHV) (serum antibody tests and/or search for DNA), JC virus (JCV) (PCR on serum), syphilis, brucellosis, coxiellosis, chlamydiosis, bartonellosis, borreliosis, mycoplasma, toxoplasmosis, histoplasmosis (serum antibody tests), and cryptococcosis (serum antigenic test). Tumoral marker and metabolic investigations were normal. Immunological investigations for lupus, Sjogren’s syndrome, type 1 diabetes, thyroiditis, and celiac disease were negative. Genetic mutations such as NOTCH3 and COL4A1 were absent.

During the same time, her son was hospitalized in the pediatrics department of our hospital for psychomotor retardation. Physical examination revealed delayed growth (weight −1.38 SD, height −2.25 SD), axial and segmental hypotonia, weak deep tendon reflexes, dystonic facial and limb movements, and psychomotor retardation (lack of head support, standing upright, grasping objects, and babbling).

Blood analyses showed microcytic, normochromic anemia (9.9 g/dL Hb, mean cell volume of 73 fL, and 32.6 g/dL mean corpuscular hemoglobin) due to iron deficiency, a normal white cell count (13.6 G/L leukocytes, 4.98 G/L neutrophil granulocytes, 0.11 G/L eosinophil granulocytes, 0.0 G/L basophil granulocytes, 7.18 G/L lymphocytes, and 1.33 G/L monocytes), normal ionogram and transaminases levels, and normal thyroid hormonal levels, but an elevated protein level (87 g/L) with hypoalbuminemia (28 g/L) and elevated IgM (7.38 g/L) and IgG (29.9 g/L) levels. CSF analyses showed lymphocytic inflammation (117 white cells/μL, 85% lymphocytes) with elevated protein (0.88 g/L), lactate (3.77 mmol/L), and IgG (0.254 g/L) levels and an elevated IgG index (0.85). Investigations for infection such as the tuberculin skin test reaction and PCR for tuberculosis in CSF were negative. Immunological investigations, such as the search for antigliadin, antiendomysium, and antinuclear antibodies were negative, and serum complement levels were normal. The search for toxins and metabolic investigations were also negative.

**Fig 2:**
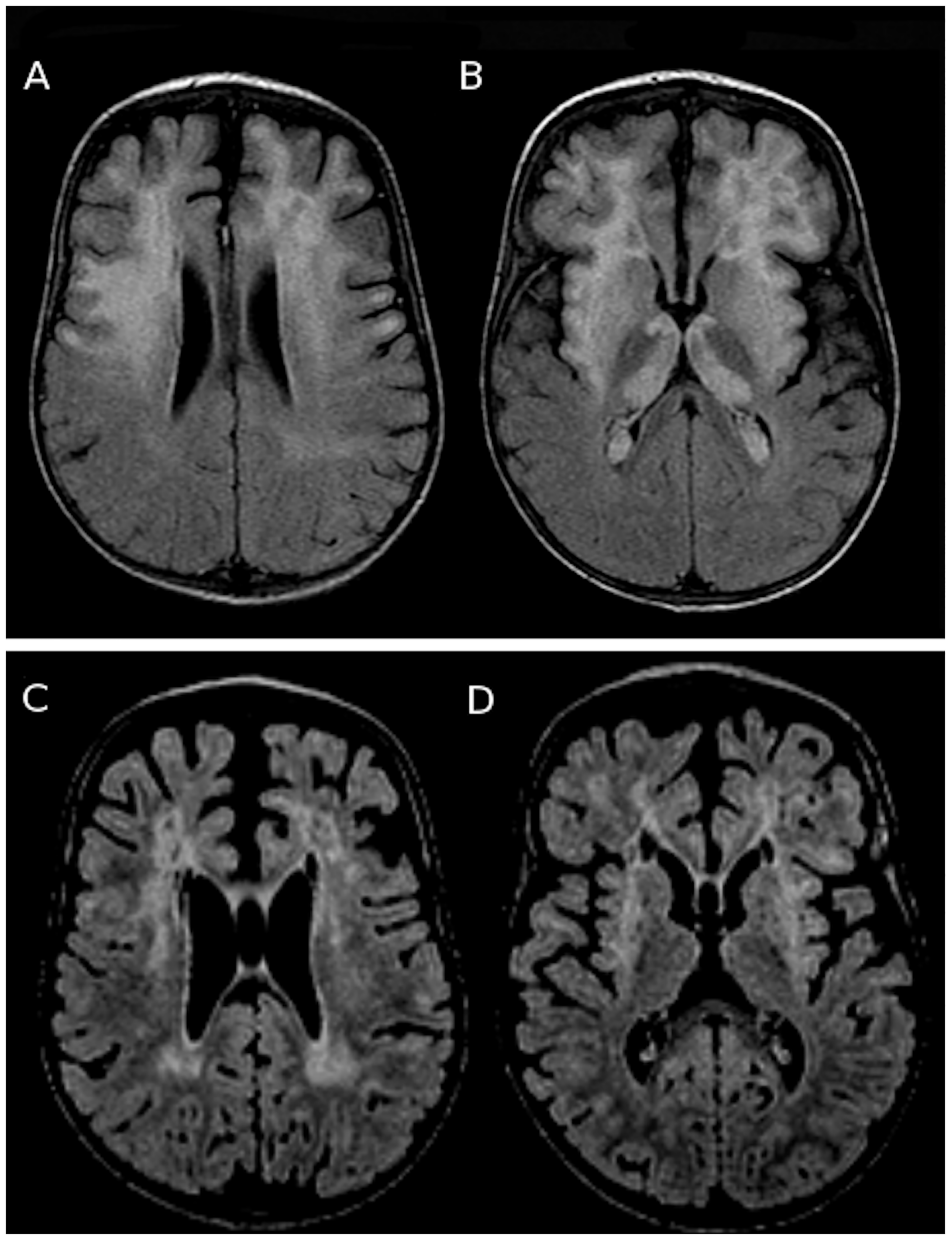
Brain MRIs of the son before and after treatment of the meningoencephalitic stage of trypanosomiasis. (A, B) Initial brain MRI in June 2013 (axial fluid attenuated inversion recovery [FLAIR]-weighted images) showing extensive and confluent hyperintensities in the frontotemporal white matter and basal ganglia (especially in the thalami and lenticular nuclei), with global brain atrophy for a 13-month-old patient. (C, D) Brain MRI in January 2014, 6 months after treatment (3D-T1 and FLAIR-weighted images), showing resolution of the thalamic lesions, diminution of the lesions in the lenticular nuclei and white matter, and increased secondary atrophy.

As with his mother, brain MRI revealed T2 hyperintensities of the frontotemporal white matter and basal ganglia, with global brain atrophy for his age (Fig 2A and 2B).

A second CSF analysis performed on the child 2 weeks later incidentally revealed motile trypanosomes on direct microscopy. Serology performed afterwards was positive for *Trypanosoma brucei gambiense* in both the serum (indirect immunofluorescence [IFI] = 100, indirect hemagglutination assay [IHA] = 32) and CSF (IFI = 0, IHA = 1).

Antibody tests performed on the mother were positive for *T*. *brucei gambiense* in both the serum (IFI = 1200, IHA = 2048) and CSF (IFI = 16, IHA = 32), but her CSF analysis did not reveal living trypanosomes (direct microscopy and CSF film) as did her blood analysis (direct microscopy, leukoconcentration, and blood film).

We diagnosed the meningoencephalitic stage of human African trypanosomiasis (HAT) for mother and son, with congenital infection for the son, as he had never entered an endemic country.

Both mother and child were treated with intravenous eflornithine (100 mg/kg/6 hours) for 2 weeks.

CSF analysis of the mother 3 weeks after treatment showed a trend towards normalization of the parameters (8 white cells/μL, 0.57 g/L protein, 3 mmol/L glucose, no trypanosomes on direct microscopy or CSF film). The search for trypanosomes in blood 8 and 22 months after treatment was negative (direct microscopy, leukoconcentration, and blood film). Follow-up serology in the serum showed a decrease of antitrypanosoma antibodies (at 3 weeks, IFI = 800, IHA = 128; at 8 months, IFI = 400; at 10 months, IFI = 100). Concerning the clinical symptoms, 1 month after treatment, pyramidal signs and clonus of the mother disappeared. After 3 years, she complained of only minor memory difficulties. Control MRIs 3 months and 1 year after the diagnosis showed diminution or even disappearance of the lesions, with increasing atrophy (Fig 1E–1H).

Concerning the child, CSF analysis 3 weeks after treatment showed a clear improvement of the parameters (29 white cells/μL, 0.31 g/L protein, 1.83 mmol/L lactate, and 3.32 mmol/L glucose). The search for trypanosomes 3 weeks after treatment was negative in blood (direct microscopy, leukoconcentration, and blood film) and CSF (direct microscopy and film), as well as in blood after 2 and 22 months. Follow-up serology of the serum showed an increase and then decrease of antitrypanosoma antibody levels (at 3 weeks, IFI = 100, IHA = 64; at 8 months, IFI = 0). Two years after treatment (3 years old) he communicated with eye contact and babbling and could grasp objects but needed permanent orthopedic appliances for severe hypotonia and enteral nutrition because of impaired deglutition. Control MRIs 1 and 6 months after diagnosis showed diminution of the lesions but increasing atrophy (Fig 2C and 2D).

## Case discussion

### Vertical transmission of HAT

HAT is caused by a parasite transmitted by the tsetse fly *(T*. *brucei gambiense* in West and Central Africa or *T*. *brucei rhodesiense* in East and southern Africa).

Congenital transmission of HAT is assumed in newborns of a HAT-infected mother where the diagnosis can be confirmed within the first 5 days of life and in older children born outside an endemic country from a mother who had acquired the infection in an endemic region [1]. Eighteen cases of certain vertical transmission of HAT have been published [1,2]; only 3 were children who never entered an endemic country themselves, born to an infected mother [3–5]. The diagnosis of these 3 cases was delayed for months (19–24 months), as in our case, probably because the symptoms were nonspecific [6] and difficult to distinguish from other causes of psychomotor retardation. The risk of vertical transmission of HAT is unknown and, although considered rare, probably underestimated [1].

### Meningoencephalitic stage of HAT

Invasion of the central nervous system occurs several months or years after infection, either through the choroid plexus or the blood brain barrier [7]. Symptoms of the meningoencephalitic stage include headache, circadian rhythm disorders, behavioral disorders, motor and/or sensitivity troubles, and extra pyramidal syndrome in cases of basal ganglia involvement [8]. This stage is more common in children, perhaps because of vertical transmission [6], and leads to death if untreated [9].

The visualization of trypanosomes in the CSF on direct microscopy, as in our case, makes the diagnosis of the meningoencephalitic stage certain, but it occurs infrequently. Parasite detection in the CSF can be improved by leukoconcentration and mini Anion Exchange Centrifugation Technique or molecular amplification techniques such as loop-mediated isothermal amplification or PCR [10]. CSF IgM can be an indirect marker, as can certain biomarkers such as chemokines or neopterin [10]. The detection of antitrypanosome antibodies in the CSF by IFI or IHA can confirm the diagnosis of the meningoencephalitic stage, even without parasite detection. In endemic regions, the World Health Organization recommends treatment if the white blood cell count in CSF is >5 cells/μL if there is suspicion of the meningoencephalitic stage, even without parasite detection [11], but this cutoff is still debated.

### Brain imaging of HAT

As noted in previous case reports [12–14], brain MRIs of the mother and her son showed extensive and confluent supratentorial and infratentorial T2 hyperintensities affecting the white matter and basal ganglia, and perivascular and leptomeningeal enhancement with diminution of these lesions after treatment (first in the basal ganglia, then in the white matter) and secondary atrophy. These abnormalities are nonspecific and may suggest many other diagnoses, such as progressive multifocal encephalopathy, acute disseminated encephalomyelopathy, metabolic leukodystrophies, lymphoma, gliomatosis, or tuberculosis [12], and may delay the diagnosis.

### Specificities of the presenting case

To our knowledge, this report is the first to provide clinical examination, brain MRI, and follow-up for both mother and child.

In our case, neurological symptoms and brain lesions were more serious in the child, and the clinical evolution and associated imaging studies showed greater disease severity following treatment than for his mother. This is likely due to the congenital infection and high level of trypanosomes and low level of antibodies in the CSF, which is harmful to the brain during development.

In summary, our report should draw the attention of neurologists and radiologists to the possibility of congenital HAT in cases of neurological symptoms, lymphocytic meningitis, and extensive brain lesions on MRI, even when a child is living in a nonendemic country and has never entered an endemic one. This diagnosis may be suspected depending on the origin of the mother and should be kept in mind by physicians as an alternative to metabolic and classic infectious diseases.

### Ethics statement

Brain MRIs and biological analysis mentioned in the case report were routine diagnostic exams. The patient gave her consent for her and her son's data to be published in a *PLOS* journal (consent form attached). We did not consult our institutional review board since the patient gave her written consent and our manuscript is a descriptive noninterventional case report. Brain MRI images are anonymized.

**Key learning points**

- HAT is possible by vertical transmission in a child who has never entered an endemic country.
- HAT should be suspected in cases of neurological and/or psychiatric symptoms with lymphocytic meningitis in patients coming from an endemic country.
- HAT is a potential diagnosis in cases of MRI brain lesions of white matter and basal ganglia in patients coming from an endemic country, in addition to metabolic, immunological, and classic infectious diseases.
- A simple serum antibody test can diagnose HAT in cases of suspect clinical symptoms and the meningoencephalitic stage can be confirmed by several methods, requiring CSF analysis.
- Despite treatment, the neurological prognosis is unfavorable in cases of delayed diagnosis of the meningoencephalitic stage of congenital HAT.
